# FoxO3 Modulates LPS-Activated Hepatic Inflammation in Turbot (*Scophthalmus maximus* L.)

**DOI:** 10.3389/fimmu.2021.679704

**Published:** 2021-07-01

**Authors:** Mingzhu Pan, Jiahuan Liu, Dong Huang, Yanlin Guo, Kai Luo, Mengxi Yang, Weihua Gao, Qiaoqing Xu, Wenbing Zhang, Kangsen Mai

**Affiliations:** ^1^ The Key Laboratory of Mariculture (Ministry of Education), The Key Laboratory of Aquaculture Nutrition and Feeds (Ministry of Agriculture and Rural Affairs), Fisheries College, Ocean University of China, Qingdao, China; ^2^ Department of Fisheries, College of Animal Science, Yangtze University, Jingzhou, China

**Keywords:** turbot, forkhead box O3, inflammation, immune, lipopolysaccharide

## Abstract

In mammals, forkhead box O3 (*foxo3*) plays important roles in liver immune system. The *foxo3* can regulate cell cycle, DNA repair, hypoxia, apoptosis and so on. However, as such an important transcription factor, few studies on *foxo3* in fish have been reported. The present study characterized the *foxo3* in turbot (*Scophthalmus maximus* L.). Lipopolysaccharide (LPS) incubated *in vitro* (hepatocytes) and injected *in vivo* (turbot liver) were used to construct inflammatory models. The *foxo3* was interfered and overexpressed to investigate its functions in liver inflammation. The open reading frame (ORF) of *foxo3* was 1998 bp (base pair), encoding 665 amino acids. Sequence analysis showed that *foxo3* of turbot was highly homologous to other fishes. Tissue distribution analysis revealed that the highest expression of *foxo3* was in muscle. Immunofluorescence result showed that *foxo3* was expressed in cytoplasm and nucleus. Knockdown of *foxo3* significantly increased mRNA levels of tumor necrosis factor-α (*tnf-α*), interleukin-1β (*il-1β)*, interleukin-6 (*il-6)*, myeloid-differentiation factor 88 (*myd88)*, *cd83*, toll-like receptor 2 (*tlr-2)* and protein level of c-Jun N-terminal kinase (JNK) in si*foxo3* + LPS (siRNA of *foxo3*+ LPS) group compared with NC + LPS (negative control + LPS) group in turbot hepatocytes. Overexpressed *foxo3* significantly decreased mRNA levels of *tnf-α*, *il-6*, nuclear transcription factor-kappa B (*nf-κb)*, *cd83*, *tlr-2* and the protein level of JNK *in vitro*. In *vivo* analysis, *foxo3* knockdown significantly increased levels of GOT in serum after LPS injection compared with NC+LPS group. Overexpressed *foxo3* significantly decreased levels of GPT and GOT in pcDNA3.1-*foxo3*+LPS group compared with pcDNA3.1+LPS group *in vivo*. *Foxo3* knockdown significantly increased mRNA levels of *tnf-α*, *il-1β*, *il-6*, *nf-κb*, *myd88* and protein level of JNK *in vivo* in si*foxo3*+LPS group compared with NC+LPS group in turbot liver. Overexpressed *foxo3* significantly decreased mRNA levels of *il-1β*, *il-6*, *myd88*, *cd83, jnk* and protein level of JNK in pcDNA3.1-*foxo3*+LPS group compared with pcDNA3.1+LPS group in turbot liver. The results indicated that *foxo3* might modulate LPS-activated hepatic inflammation in turbot by decreasing the proinflammatory cytokines, the levels of GOT and GPT as well as activating JNK/caspase-3 and *tlr-2/myd88/nf-κb* pathways. Taken together, these findings indicated that FoxO3 may play important roles in liver immune responses to LPS in turbot and the research of FoxO3 in liver immunity enriches the studies on immune regulation, and provides theoretical basis and molecular targets for solving liver inflammation and liver injury in fish.

## Introduction

The forkhead O (FoxO) family has a highly conserved amino acid sequence called the forkhead domain (FD). Transcription factors of FoxO subfamily include FoxO1, FoxO3, FoxO4 and FoxO6 in mammals. Members of the FoxO family regulate a variety of cellular functions, such as cell differentiation, metabolism, proliferation and apoptosis (1, 2). Many studies have shown that FoxO is an essential regulator of different liver diseases (3). Mice that are deficient in FoxO1/3/4 specifically in hepatocytes are sensitive to high-fat cholesterol diet-induced inflammation and liver injury (4). FoxO1/3/4 in hepatocytes play a protective role in diet-induced liver fibrosis. The role of FoxOs in the modulation of immune response and inflammation is essential (5). In the studies of mice, lipopolysaccharide (LPS) administration decreased FoxO activity and increased nuclear transcription factor-kappa B (NF-κB) activity (6). In general, the FoxO family plays a crucial role in immunity.

FoxO3 is one of the most important members of the FoxO family. In mammals, FoxO3 has been found to be a multifunctional transcription factor involved in regulating gene expression in the cell cycle, DNA repair, hypoxia and apoptosis (7–12). In mice, overexpression of FoxO3 improves inflammatory status in by decreasing the levels *il-1β*, *il-6*, *caspase-3* and other genes (13). Interleukin-1β (*il-1β*), interleukin-6 (*il-6*) and tumor necrosis factor-α (*tnf-α*) are pro-inflammatory cytokines that are the major markers of inflammation (14). The liver is a main metabolic and alexipharmic organ and is vital for host defense and tissue repair in infection (15). In the study of mice liver, it was reported that overexpression of FoxO3 attenuated liver failure by downregulating the expressions of *tnf-α*, *il-1β* and *il-6* (16). In addition, it was demonstrated that FoxO3 protected the liver from ethanol-induced inflammation in mice (17). Based on the data above, FoxO3 plays a vital role in liver immune regulation.

Although many studies on FoxO3 have been carried out in mammals, research of FoxO3 in fish is still severely lacking. It was found that the mitochondrial autophagy pathway in liver of large yellow croaker (*Larimichthys crocea*) was regulated by the FoxO3 (18). Dietary L-leucine attenuated the protein degradation *via* regulating the protein kinase B (AKT)/FoxO3 signaling pathway in hybrid catfish (*Pelteobagrus vachelli × Leiocassis longirostris*) (19). In addition, it was reported that the expression *foxo3* was significantly upregulated in channel catfish (*Ictalurus punctatus*) after bacterial infection (20). Hence, it is necessary to investigate the molecular immune mechanism of *foxo3* in fish.

Turbot (*Scophthalmucs maximus* L.) is one of the most commercially important marine fish species, which was widely cultured in Europe and Asia (21). Turbot culture represents a large proportion of land-based tank-cultured fish in China, especially in recirculating aquaculture systems. What’s more, turbot industries worldwide have suffered heavily from disease outbreaks caused by bacterial infections including *Edwardsiella tarda* and different species of *Vibrio*, such as V. *anguillarum* (22–24). These caused huge economic losses to the turbot industry. Therefore, it is indispensable to study the molecular immune mechanism of turbot. There were few studies on *foxo3* in turbot, and the only report was that *foxo3* belongs to chemokine signaling pathway enriched in omics study of turbot (25). The role of *foxo3* in the immune of turbot is unclear. LPS is found in the outer membrane of Gram-negative bacteria. Recent evidences from different animal models indicate that LPS-induced acute liver injury (26). Therefore, LPS incubated *in vitro* and injected *in vivo* were used to construct liver inflammatory models to determine whether *foxo3* regulates immune signaling pathway in turbot liver. It helps to improve the understanding of the functions of *foxo3* in turbot even in carnivorous fish.

## Materials and Methods

The protocols for animal care and handing used in this study were approved by the Institutional Animal Care and Use Committee of Ocean University of China.

### Animals

Turbots (*Scophthalmus maximus* L.) (10.0 ± 0.5 g) were purchased from a commercial farm (Yantai, Shandong Province, China) and maintained at 20 ± 1°C in aerated seawater. The fish were fed with a commercial feed (crude protein: 48%; crude lipid: 10%) twice daily and acclimated to the laboratory setting for two weeks before all the experiments.

### Molecular Cloning of Turbot Foxo3 and Sequence Analysis

The cloning of turbot *foxo3* was performed by slightly modifying the previously described methods (21). Total mRNA was extracted from turbot liver using Trizol Reagent (Invitrogen, USA). The quality and concentration of RNA were measured by Nanodrop 2000 (Thermo Fisher Scientific, USA). Then, the quality-compliant RNA (1000 ng) was reversely transcribed into cDNA using the PrimeScript™ RT reagent Kit with gDNA Eraser (RR047A, Takara, Japan), and amplification systems and procedures were performed according to the manufacturer’s instructions. PCR primers for CDS-*foxo3* cloning were designed as in **Table 1**. The PCR amplification system was performed with the following parameters: initial denaturation at 95°C for 2 min and 35 cycles of 95°C for 5 s, 55°C for 30s and 72°C for 1 min, followed by 72°C for 10 min. The PCR product was analyzed in 1.2% agarose gel electrophoresis, purified, and ligated to the pEASY-Blunt cloning vector (CB101-01, TransGen, China). Then, the vector was transformed into Trans1-T1 Phage Resistant Chemically Competent Cell (CD501-02, TransGen, China). The PCR products were sequenced in Sangon Biotech (Shanghai, China).

**Table 1:** Sequences of the qPCR primers.

Primers	Sequence (5’-3’)	GenBank Accession Number
*CDS-foxo3-*F	ATGGCCGAGGCGCAGCTGGC	MW365446
*CDS-foxo3-*R	TCAGCCAGGTACCCTCAGCCAGGT	
*RT-foxo3-*F	TACTTCAAGGACAAAGGCGACAG	MW365446
*RT-foxo3-*R	CATTGGAGTTAGTGCGGGAGC	
*tnfα*-F	GGGTGGATGTGGAAGGTGAT	FJ654645.1
*tnfα*-R	GGCCTCTGTTTGGCTTGACT	
*il-1β*-F	GCGACATGGTGCGATTTCTG	AWP07584.1
*il-1β*-R	GCTGGATGCTGAAGGTCTGG	
*il-6*-F	GCCATGAAGAAGTGTCGG	AWP21758.1
*il-6*-R	TTGTGGTTGCTGGAGGG	
*nf-κb*-F	ACACTGCTGAGCTGAAGATC	MF370855
*nf-κb*-R	CTCTGAGCCCATCAGGGTC	
*myd88*-F	CCCAATGGTAGCCCTGAGAT	KP985236
*myd88*-R	CATCTCGGTCGAACACACAC	
*cd83*-F	AGTACTACGTCGGCTTGGAC	HM159997.1
*cd83*-R	CTGTCACAGTGAGGAGGACC	
*jnk*-F	CTGGTAGAGCAGGTAGGACA	SRP172919
*jnk*-R	CACAGAAGACACTGGAAGAA	
*tlr-2*-F	AGTCTGTTCAGCCGTTCAGA	KU746963.1
*tlr2*-R	CTCGTCGATGCTCCTCTGAT	
*β-actin*-F	GTAGGTGATGAAGCCCAGAGCA	EU686692.1
*β-actin*-R	CTGGGTCATCTTCTCCCTGT	

foxo3, forkhead box o3; tnfα, tumor necrosis factor-α; il-1β, interleukin-1β; il-6, interleukin 6; nf-κb, nuclear factor κB; myd88, myeloid-differentiation factor 88; jnk, c-Jun N-terminal kinase; tlr-2, toll-like receptor 2.

The methods of sequence analysis and phylogenetic tree construction were described in the previous study (21). In short, the phylogenetic tree was conducted based on multiple sequence alignments using the Neighbor-Joining method in the MEGA 5.0 program.

### Tissue Distribution

Three turbots were used and 12 tissues (skin, eye, liver, gill, brain, stomach, head kidney, intestine, adipose, spleen, heart and muscle) were isolated to analyze the *foxo3* expression level in different tissues. Samples were kept stored at -80°C until analysis.

### Immunofluorescence

The immunofluorescence was used to analyze the distribution of FoxO3 in hepatocytes. In brief, a piece of liver tissue was taken out from a turbot, then washed with PBS and fixed in 4% paraformaldehyde at room temperature for 30 min. Then, it was blocked for 1 h in 3% BSA, and followed by incubation with specific primary monoclonal rabbit anti-human antibody FoxO3 (1:200, AF609, Beyotime, China) (diluted with PBS appropriately) overnight at 4°C. The suitability of the antibody FoxO3 for turbot has been verified prior to use (**Supplementary Figures 1, 2**). After that, the tissue was washed for three times with PBS for 5 min each time, followed by incubation with secondary antibody (Cy3 conjugated Goat Anti-Rabbit IgG) (1:200, GB21303, Servicebio, China) for 60 min at room temperature in the dark. Microscopy detection and images collection were by fluorescent microscopy (Eclipse C1, Nikon, Japan). The nucleus is blue by labeling with DAPI. Positive cells are red according to the fluorescent labels used.

### The Synthesis of siRNA and Its Knocking Down Efficiency

Four siRNA duplexes (siRNA-485, siRNA-783, siRNA-1071, siRNA-1349, respectively) targeting different encoding regions of *foxo3* and a silence negative control siRNA (si*foxo3*-NC) were designed and synthesized by Sangon (Sangon Biotech, China) (**Table 2**).

**Table 2:** SiRNA sequences.

Primer	Sense (5’-3’)	Antisense (5’-3’)
si*foxo3*-485	GAGGTCCATCCCCTACTTCAA	UUGAAGUAGGGGAUGGACCUCTT
si*foxo3*-783	GACAGTCCTTCAGGTCTCTCT	AGAGAGACCUGAAGGACUGUCTT
si*foxo3*-1071	GGTACGATGAACCTGAATGAT	AUCAUUCAGGUUCAUCGUACCTT
si*foxo3*-1349	GCAGACCATCCAGGAGAACAA	UUGUUCUCCUGGAUGGUCUGCTT
si*foxo3*-NC	UUCUCCGAACGUGUCACGUTT	ACGUGACACGUUCGGAGAATT

Primary hepatocytes were isolated from turbot liver and cultured as described previously (21). Cells were seeded in 6-well plates (Corning, USA) at a density of 1.0 × 10^6^ cells/well and incubated at 24°C. When the cells were at 80–90% confluency, they were transfected with siRNAs. For transfection, 5 μg of siRNA and 3.75 μl of Lipofectamine 3000 reagent (L3000015, Invitrogen, USA) were used in each well according to the manufacturer’s protocol. Scrambled siRNA (si*foxo3*-NC) served as a negative control for the experiment. No siRNA but an equal amount of PBS (Sangon Biotech, China) was added as a control group. Cells were harvested after 24 h to determine transfection efficiency by qPCR. The transfection experiments were performed in triplicate. Experiments would be subsequently performed to assess the silence effects only if the transfection effects were > 50%.

### Plasmid Construction

A PCR product encoding the entire open reading frame (ORF) of *foxo3* was cloned into the expression vector pcDNA3.1 (Invitrogen, USA). A single band of 1998 bp corresponding to the expected amplification product was obtained from 2.2. The template of *foxo3* was PCR amplified using primers with homology arms to BamHI region in pcDNA3.1 consisting of the forward primer *foxo3*-bamh1-F: 5’- cttggtaccgagctcggatccATGGCCGAGGCGCAGCTG-3’ and the reverse primer *foxo3*-bamh1-R: 5’-atggtggcgaccggtggatccGCCAGGTACCCAGCTCTGAGA-3’. The resulting amplicons were purified by gel electrophoresis and extracted using the SanPrep Column DNA Gel Extraction Kit (B110092, Sangon Biotech, China). The pcDNA3.1 plasmid was digested with enzyme BamHI (D1010A, Takara, Japan). The purified resulting amplicons was then ligated into the pcDNA3.1 plasmid using ClonExpress Ultra One Step Cloning Kit (C112-02, Vazyme, China). The plasmid constructed was called pcDNA3.1-*foxo3*, and construct was confirmed by DNA sequencing. The pcDNA3.1-*foxo3* plasmid was then transformed into Trans1-T1 Phage Resistant Chemically Competent Cell. After shake culture overnight, adequate plasmid for transfection was collected using a EasyPure HiPure Plasmid MaxiPrep Kit (EM121-01, TransGen, China).

### MTT Assay for Cell Viability

For the determination of cell viability, primary hepatocytes were seeded in 96 well plates and treated with different kinds of treatments below. A MTT assay was performed to test cell viability according to standard protocols (M1020, Solarbio, China).

### LPS Stimulating and Knocking Down of Foxo3 *In Vitro* and *In Vivo*

To study the effects of *foxo3* in the biological behavior of LPS-activated hepatocellular inflammation, turbot primary hepatocytes were divided into three groups in 6 well plates: (1) NC: cells treated with one scrambled siRNA (5 μg/well); (2) NC + LPS: cells treated with the scrambled siRNA (5 μg/well) and LPS (100 ng/ml); (3) si*foxo3* + LPS: cells treated with the si*foxo3* (5 μg/well) and LPS (100 ng/ml). Cells were harvested after 24 h incubation and stored at -80 °C for qPCR and Western Blot.

Injection test was used to examine the efficiency of the siRNA *in vivo*. The injection dose was determined by fish body weight. Two groups of turbot (12 fish/group) were intraperitoneally injected with NC (1 μg/g) and si*foxo3* (1 μg/g), respectively. At 48 h post-injection, the expression of *foxo3* in the liver was determined by RT-qPCR to guarantee *foxo3* was knocked down successfully.

Thirty-six turbots were divided into three groups (12 fish/group): (1) NC: turbots intraperitoneally injected with NC (1 μg/g) and PBS (100 μl); (2) NC + LPS: turbots intraperitoneally injected with NC (1 μg/g) and LPS (2.5 μg/g); (3) si*foxo3* + LPS: turbots intraperitoneally injected with the *sifoxo3* (1 μg/g) and LPS (2.5 μg/g). All the injection doses above were determined by fish body weight. The NC and si*foxo3* were injected 24 h before injecting PBS and LPS. When turbots were intraperitoneally injected with PBS/LPS 24 h later, the serum and liver of turbots were collected and immediately frozen in liquid nitrogen. Three turbots were mixed together to make a sample.

### LPS Stimulating and Overexpression of Foxo3 *In Vitro* and *In Vivo*

Turbot primary hepatocytes were seeded in 6-well plates at a density of 1.0 × 10^6^ cells/well and incubated at 24 °C. When the hepatocytes were at 80–90% confluency, the cells were transfected with pcDNA3.1 or pcDNA3.1-*foxo3* using Lipofectamine 3000 (L3000015, Invitrogen, USA) according to manufacturer’s instruction. For each well, 5 μg plasmid was used. Meanwhile, LPS was added into the wells after the plasmid transfection. There were three treatments respectively: (1) pcDNA3.1: cells treated with pcDNA3.1 (5 μg/well); (2) pcDNA3.1 + LPS: cells treated with pcDNA3.1 (5 μg/well) and LPS (100 ng/ml); (3) pcDNA3.1-*foxo3* + LPS: cells treated with the pcDNA3.1-*foxo3* (5 μg/well) and LPS (100 ng/ml). Cells were harvested after 24 h incubation and stored at -80 °C for RT-qPCR and Western Blot.

Prior to the overexpression of *foxo3 in vivo*, a pre-test was conducted to determine whether the plasmid could enter into liver of turbot intraperitoneally injected with pcDNA3.1-EGFP (1 μg/g) (**Supplementary Figure 3**). Injection dose was determined by fish body weight. After that, to examine the efficiency of the pcDNA3.1-*foxo3 in vivo*, two groups of turbot (12 fish/group) were intraperitoneally injected with pcDNA3.1 (1 μg/g) and pcDNA3.1-*foxo3* (1 μg/g), respectively. At 48 h post-injection, the expression of *foxo3* in the liver was determined by RT-qPCR to guarantee *foxo3* was upregulated successfully.

Thirty-six turbots of experiment were divided into three groups (12 fish/group): (1) pcDNA3.1, turbots intraperitoneally injected with pcDNA3.1 (1 μg/g) and PBS (100 μl); (2) pcDNA3.1 + LPS: turbots intraperitoneally injected with pcDNA3.1 (1 μg/g) and LPS (2.5 μg/g); (3) pcDNA3.1-*foxo3* + LPS: turbots intraperitoneally injected with the pcDNA3.1-*foxo3* (1 μg/g) and LPS (2.5 μg/g). All the injection doses above were determined by fish body weight. The pcDNA3.1 and pcDNA3.1-*foxo3* were intraperitoneally injected 24 h before injecting PBS and LPS. When turbots were intraperitoneally injected with PBS/LPS 24 h later, the serum and liver of turbots were collected and immediately frozen in liquid nitrogen. Three turbots were mixed together to make a sample.

### Serum Aminotransferase Activities

Plasma samples were taken from the turbots at 24 h after PBS/LPS injection described at 2.8 and 2.9. Serum levels of GPT (C009-2-1; Jiancheng, China), GOT (C010-2-1; Jiancheng, China) as markers of hepatic damage were measured performed according to the manufacturer’s instructions.

### Quantitative Real-Time PCR (qPCR)

The expression of target genes including *foxo3*, *tnfα*, *il-1β*, *il-6*, *nf-κb*, *myd88*, *cd83*, *jnk* and *tlr2* were detected according to the method described previously (21). Briefly, total RNA from samples was extracted with Trizol Reagent (Invitrogen, USA). The quality and quantity of RNA were assessed using agarose gel electrophoresis at 1.2% and spectrophotometric analysis (A 260:280 nm ratio). After that, complementary DNA (cDNA) was synthesized from 1 mg of total RNA using the PrimeScript™ RT reagent Kit with gDNA Eraser (RR047A, Takara, Japan). First strand cDNA was diluted 3 times using sterilized double-distilled water. qPCR was carried out in a quantitative thermal cycle (Quant Studio 5, Applied Biosystems, USA). The final reaction mixture contained 15 μL containing 7.5 μL 2 × SYBR Green Realtime PCR Master Mix (Q711-02, Vazyme Biotech, China), 0.3 μL each of primers (10 μmol/L) and 1 μL of cDNA. *β-actin* was used as an endogenous reference to normalize the template amount. Real-time PCR temperature profile was 95°C for 30 s followed by 40 cycles of 10 s at 95°C, 30 s at 58°C. To confirm the specificity and purity of all PCR products, melt curve analysis was carried out after amplification: 95°C for 15 s, 60°C for 1 min and 95°C for 1 s. The gene expressions were determined using the 2^−ΔΔCt^. All the primers used in this study were listed in **Table 1**.

### Western Blot Analysis

Protein homogenates preparation from turbot liver and hepatocytes, and western blotting were processed as refer to previous studies (27, 28). Equal amounts (30 μg per lane) of protein were separated by sodium dodecyl sulfate-polyacrylamide gels (SDS-PAGE). Gels were cut according to molecular weight and transferred to 0.45 μm PVDF membrane (Millipore, Billerica, MA, USA). Different target proteins were tested in independent membrane, respectively. Incubation with the primary antibody was performed overnight at 4°C. The primary antibodies used were polyclonal antibody rabbit anti-human JNK (1:500, WL05246, WanleiBio, China), polyclonal antibody rabbit anti-human cleaved-caspase-3 (1:500, WL02117, WanleiBio, China) and the polyclonal rabbit anti-human internal antibody control glyceraldehyde-3-phosphate dehydrogenase (GAPDH, 1:1000, AB-P-R001, Goodhere Biotechnology, China). After the incubation, the membrane was washed with TBST and incubated with secondary antibody (HRP-labeled goat anti-Rabbit lgG) (A0208, Beyotime, China) at 1:5000 dilution for 1 h at room temperature. The immune complexes were visualized using ECL reagents (P0018FM, Beyotime, China). The band densities were quantified using ImageJ software (National Institutes of Health, Bethesda, MD, USA). The suitability of the antibodies (JNK, cleaved-caspase-3 and GAPDH) for turbot has been verified prior to use (**Supplementary Figure 4**).

### Statistical Analysis

All data were presented as means ± S.D. (standard deviation) and showed all the individual data points. All statistical analyses were performed using SPSS 20 (IBM, IL, USA). The silencing efficiency of the siRNAs (si*foxo3*-NC and si*foxo3*-1349) and the overexpression efficiency of *foxo3* (pcDNA3.1 and pcDNA3.1-*foxo3*) *in vitro* and *in vivo* were analyzed by t-test. Other statistical evaluations were analyzed by one-way analysis of variance (ANOVA) followed by Tukey’s multiple range tests. *P*-value < 0.05 was considered to be statistically significant.

## Results

### Cloning, Characterization, Sequence Analysis and Tissue Distribution of Turbot Foxo3

**Figure 1:**
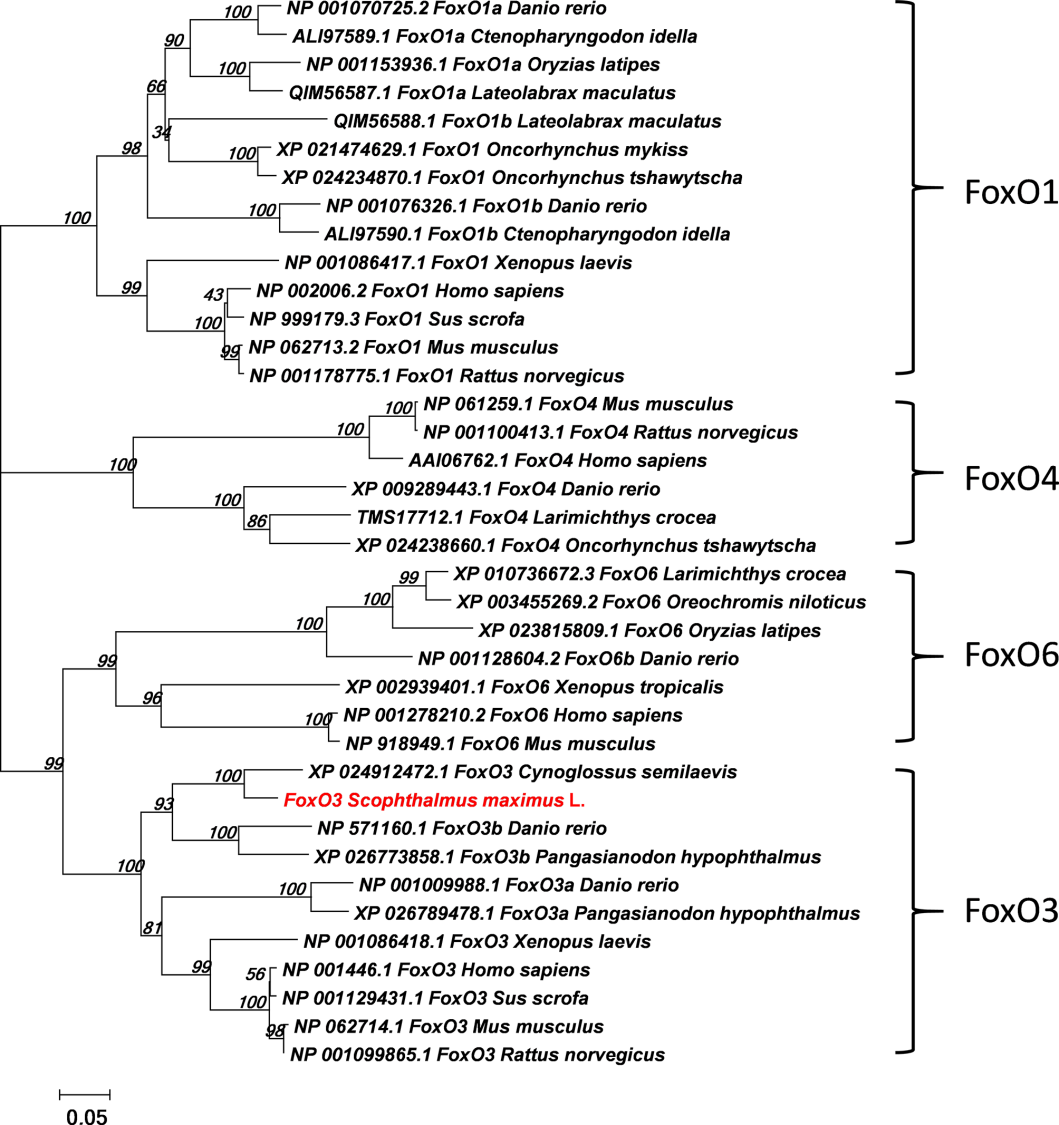
Phylogenetic tree comparing turbot (*Scophthalmus maximus* L.) *foxo* with amino acid sequences from other species. The phylogenetic tree was constructed using the Neighbor-Joining method in MEGA5. The branch length is proportional to the amino acid substitution rate per site. The numbers represent the frequencies (%) with which the tree topology presented was replicated after 1000 iterations.

**Figure 2:**
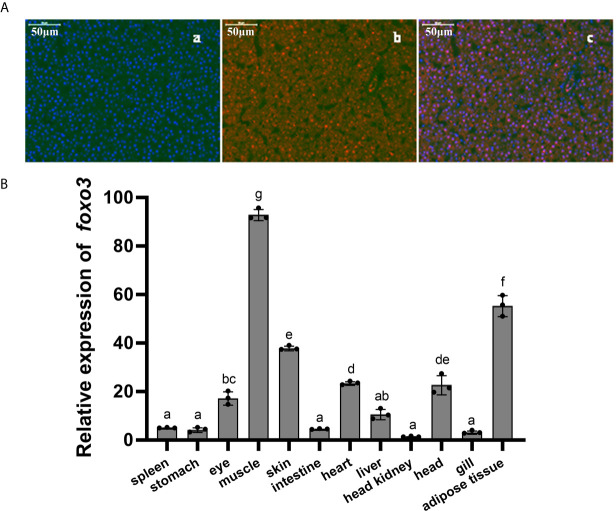
**(A)** Immunofluorescent localization of FoxO3 in turbot liver. (a) DAPI staining; (b) FoxO3 staining; (c) Merge. **(B)** Expressions of *foxo3* in different tissues of turbot. The results were expressed as the mean ± standard deviation (n = 3). Bars bearing the different letters are significantly different between tissues by one-way analysis of variance (ANOVA) (*P*< 0.05).

The full-length ORF of *foxo3* was 1998 bp (GenBank Accession No: MW365446), encoding peptides of 665 amino acids with a predicted molecular weight of 70.06 kDa and a theoretical isoelectric point of 4.80. The phylogenetic tree was constructed with the full-length *foxo* amino acid of various species of *foxo1*, *foxo3*, *foxo4* and *foxo6* (**Figure 1**). The most similar species was tongue soles (*Cynoglossus semilaevis*). The immunofluorescent results showed that the FoxO3 expressed both in cytoplasm and nucleus (**Figure 2A**). The *foxo3* was broadly distributed in various tissues, and the abundance of mRNA varied among different tissues (**Figure 2B**). The expression level of *foxo3* gene was highest in the muscle, followed by adipose tissue, skin, heart, head, eye, liver, spleen, intestine, stomach, gill and head kidney, respectively. The gene expression of *foxo3* in head kidney was the lowest relatively.

### Inflammation-Related Genes and Proteins With Foxo3 Knocked Down *In Vitro*

**Figure 3:**
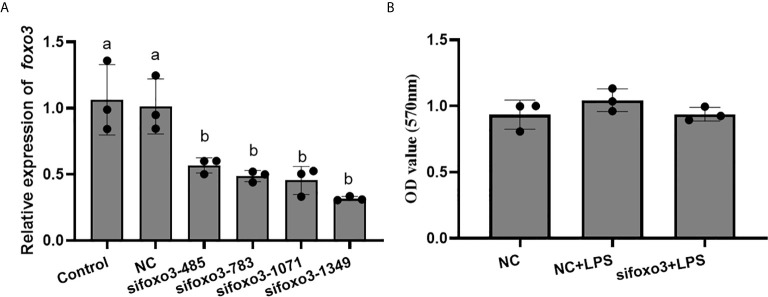
**(A)** Relative level of *foxo3* mRNA in primary cultured hepatocytes treated with si*foxo3*. Results are represented as mean ± standard deviation (n = 3). Values with different letters mean significant differences (*P* < 0.05). **(B)** The OD values of MTT. Results are represented as mean ± standard deviation (n = 3). Values with different letters mean significant differences (*P* < 0.05).

The result indicated that si*foxo3*-1349 was the most efficient duplex for knocking down *foxo3* expression in turbot liver cells (decreasing about 60%) (**Figure 3A**), and this siRNA duplex was subsequently delivered in the following experiments. The cell viability was detected by MTT assay in the three groups. The result showed that there was no significant difference among the three groups (*P* > 0.05) (**Figure 3B**).

**Figure 4:**
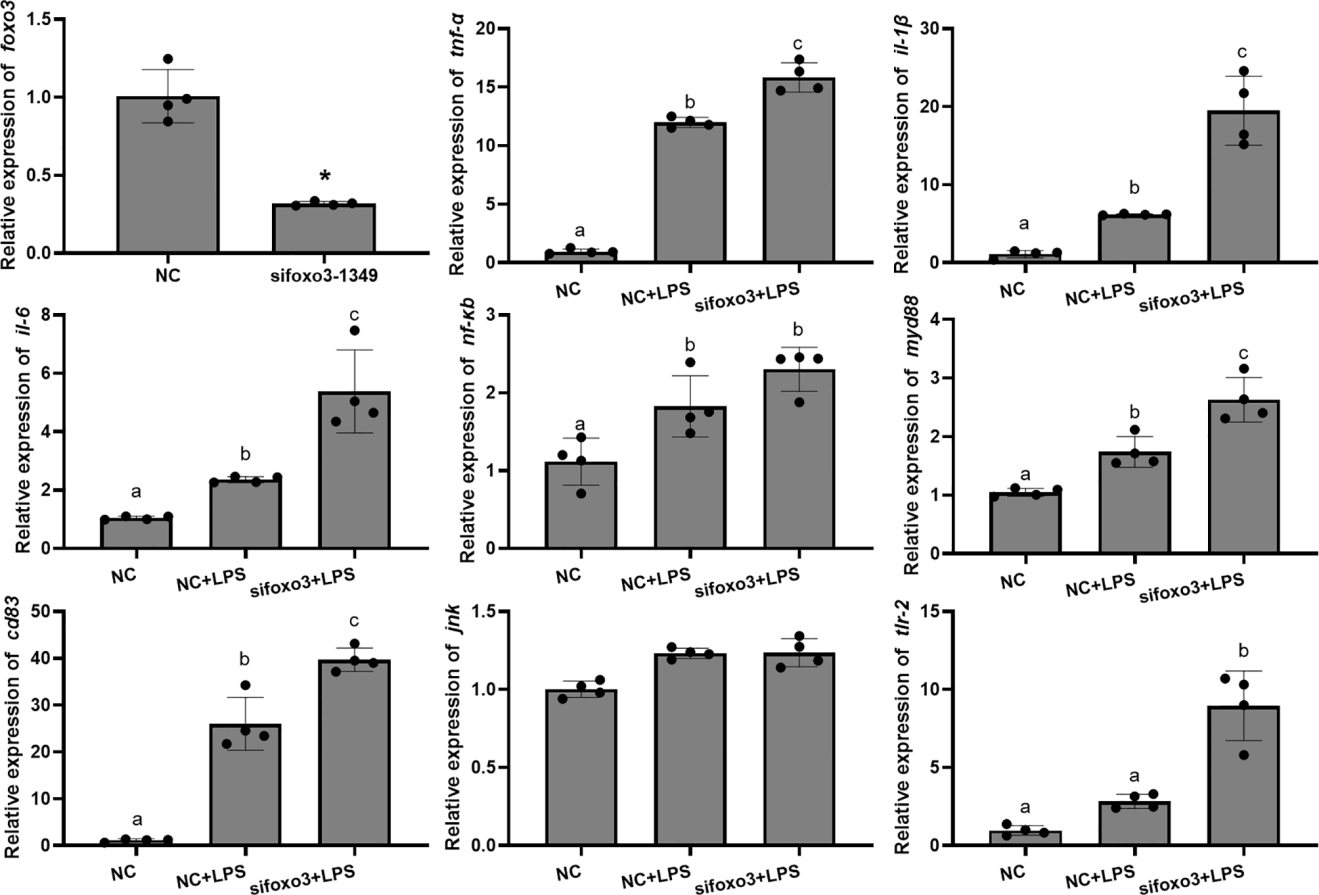
Relative mRNA levels of *foxo3*, *tnf-α*, *il-1β*, *il-6*, *nf-κb*, *myd88*, *cd83*, *jnk* and *tlr-2* in primary hepatocytes of turbot. The results are expressed as the mean ± standard deviation (n = 4). ‘*’ means significantly different between two group (*P* < 0.05). The different letters are significantly different (*P* < 0.05).

The mRNA levels of *tnf-α*, *il-1β*, *il-6*, *nf-κb*, *myd88* and *cd83* significantly increased after LPS incubation compared with the NC group (*P* < 0.05). The mRNA level of *jnk* and *tlr-2* increased after LPS incubation compared with the NC group, but there was no significant difference (*P* > 0.05). The mRNA levels of *tnf-α*, *il-1β*, *il-6*, *myd88*, *cd83* and *tlr-2* significantly increased in the si*foxo3*+LPS group compared with NC+LPS group (*P* < 0.05). The mRNA levels of *nf-κb* and *jnk* increased in the si*foxo3*+LPS group compared with the NC+LPS group, but there was no significant difference between the two groups (*P* > 0.05) (**Figure 4**).

**Figure 5:**
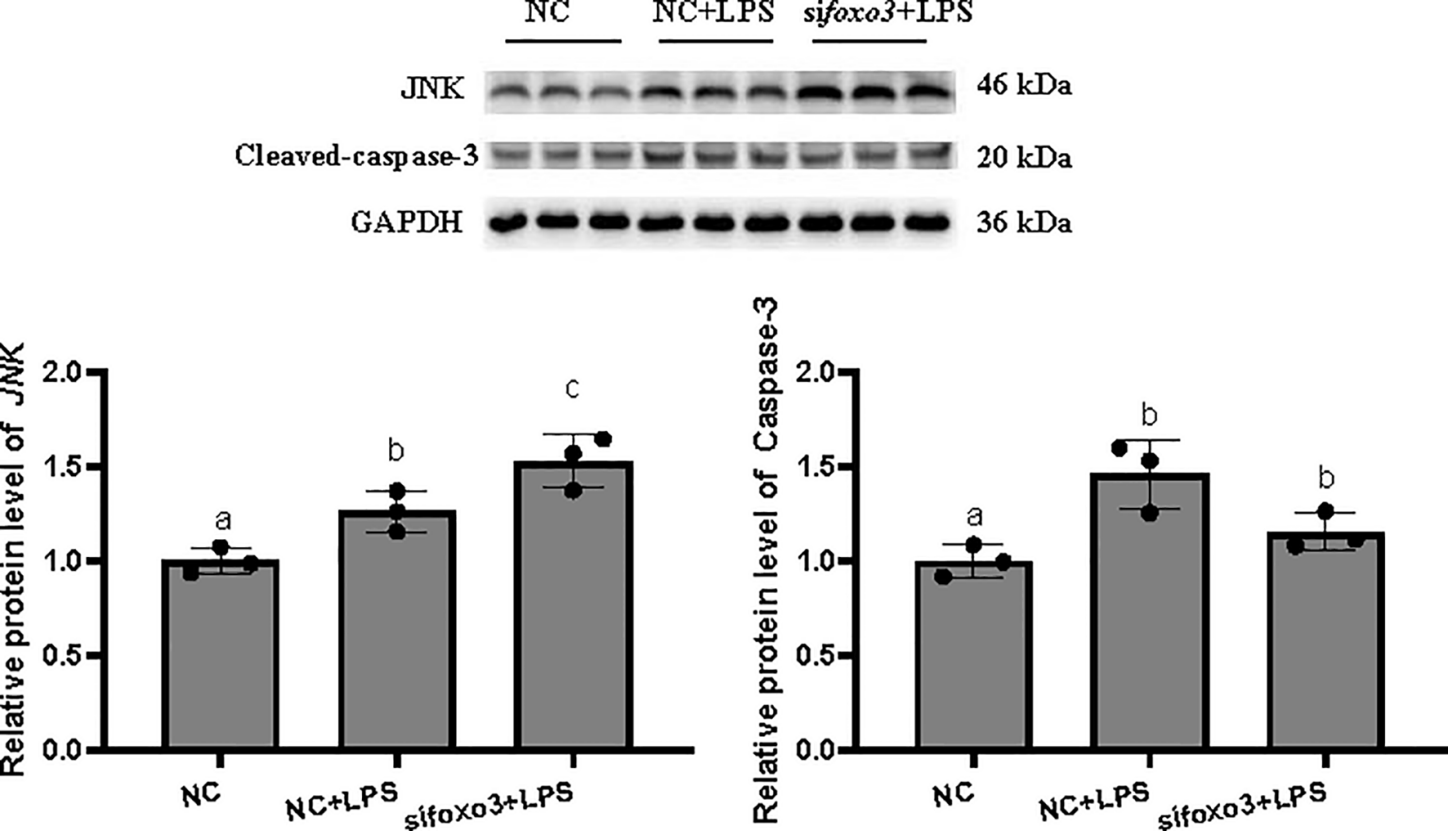
Protein levels in turbot hepatocytes. The relative protein abundances of JNK and cleaved-caspase-3 in hepatocytes were measured by western blot and expressed as relative expression values to those in NC group. Values are represented as the mean ± standard deviation (n = 3). Values with different letters mean significantly difference (*P* < 0.05).

The protein levels of JNK and cleaved-caspase-3 significantly increased after LPS incubation compared with the NC group (*P* < 0.05). The protein level of JNK significantly increased in si*foxo3*+LPS group compared with the NC+LPS group (*P* < 0.05) and the protein level of cleaved-caspase-3 had no difference between the two groups (*P* > 0.05) (**Figure 5**).

### Inflammation-Related Genes, Enzyme Activity and Proteins With Foxo3 Knocked Down *In Vivo*

**Figure 6:**
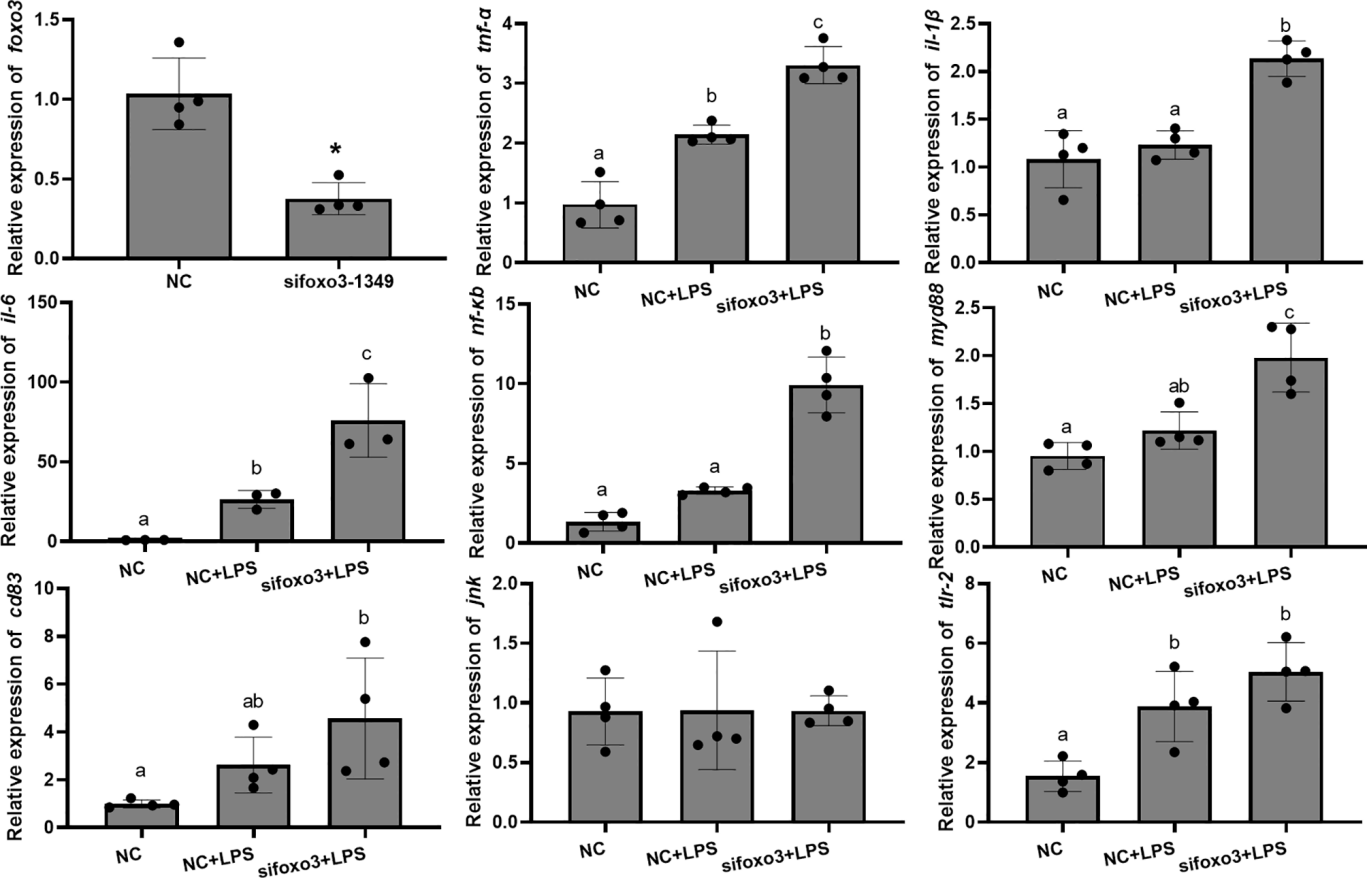
Relative mRNA expression of *foxo3*, *tnf-α*, *il-1β*, *il-6*, *nf-κb*, *myd88*, *cd83*, *jnk*, *tlr-2* in turbot liver. The results are expressed as the mean ± standard deviation (n = 4). ‘*’ means significantly different between two group (*P* < 0.05). The different letters are significantly different (*P* < 0.05).

To investigate the involvement of FoxO3 in LPS-induced liver inflammation *in vivo*, turbots were injected with specific small interfering *in vivo*. The result indicated that si*foxo3*-1349 knocked down *foxo3* expression in turbot liver cells (decreasing > 50%) (**Figure 6**). The mRNA levels of *tnf-α*, *il-6*, and *tlr-2* significantly increased after LPS injection compared with NC group (*P* < 0.05). The mRNA levels of *il-1β*, *nf-κb*, *myd88* and *cd83* increased after LPS injection compared with NC group, but there was no significant difference (*P* > 0.05). The mRNA levels of *tnf-α*, *il-1β*, *il-6*, *nf-κb* and *myd88* significantly increased in si*foxo3*+LPS group compared with NC+LPS group (*P* < 0.05). The mRNA levels of *cd83* and *tlr-2* increased in the si*foxo3*+LPS group compared with the NC+LPS group, but there was no significant difference between the two groups (*P* > 0.05). The mRNA levels of *jnk* remained unchanged among the three groups (*P* > 0.05) (**Figure 6**).

**Figure 7:**
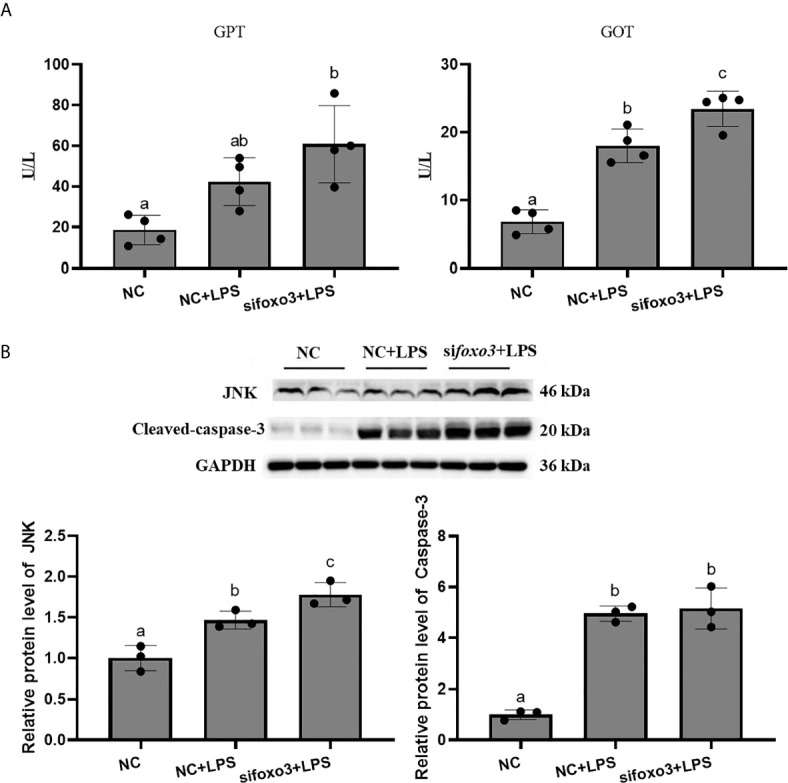
**(A)** The levels of glutamate pyruvate transaminase (GPT) and glutamate oxaloacetate transaminase (GOT) in serum. Values are represented as the mean ± standard deviation (n = 4). Values with different letters mean significantly difference (*P* < 0.05). **(B)** Protein levels in turbot liver. The relative protein abundances of JNK and cleaved-caspase-3 in turbot liver were measured by western blot and expressed as relative expression values to those in NC group. Values are represented as the mean ± standard deviation (n = 3). Values with different letters mean significantly difference (*P* < 0.05).

Results of GPT and GOT in serum showed that the level of GOT significantly increased after LPS injection compared with the NC group (*P* < 0.05). The level of GPT increased after LPS injection compared with the NC group. There was no significant difference between the two groups (*P* > 0.05). The level of GOT in serum significantly increased in si*foxo3*+LPS group compared with NC+LPS group (*P* < 0.05) (**Figure 7A**). The level of GPT in serum increased in si*foxo3*+LPS group compared with NC+LPS group, but there was no significant difference between the two groups (*P* > 0.05) (**Figure 7A**).

The protein levels of JNK and cleaved-caspase-3 significantly increased after LPS injection compared with NC group (*P* < 0.05). The protein levels of JNK significantly increased in si*foxo3*+LPS group compared with NC+LPS group (*P* < 0.05) and the protein level of cleaved-caspase-3 had no difference between the two groups (*P* > 0.05) (**Figure 7B**).

### Inflammation-Related Genes and Proteins With foxo3 Overexpressed *In Vitro*

**Figure 8:**
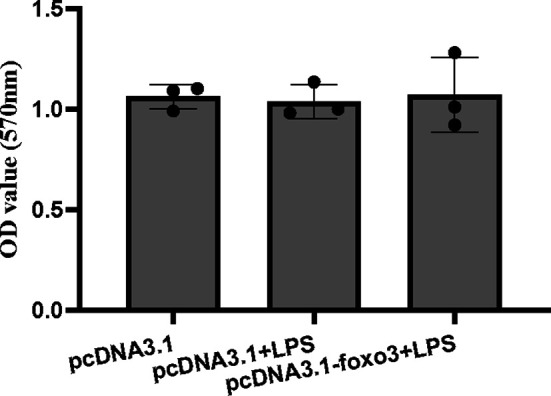
The OD values of MTT. Results are represented as mean ± standard deviation (n = 3). Values with different letters mean significant differences (*P* < 0.05).

The MTT result showed that there was no significant difference among the pcDNA3.1 group, pcDNA3.1+LPS group and pcDNA3.1-*foxo3+*LPS group (*P* > 0.05) (**Figure 8**).

**Figure 9:**
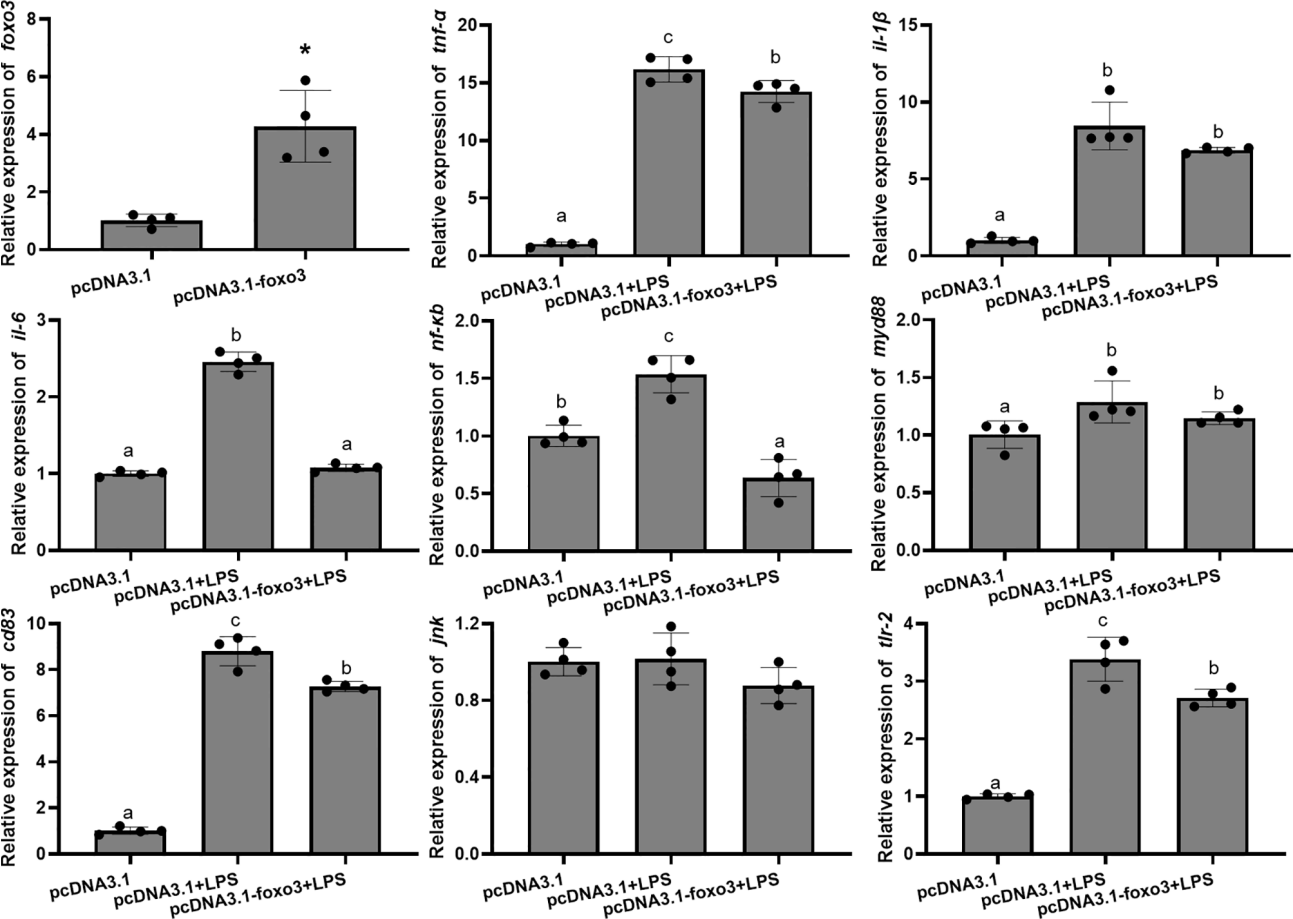
Relative mRNA levels of *foxo3*, *tnf-α*, *il-1β*, *il-6*, *nf-κb*, *myd88*, *cd83*, *jnk* and *tlr-2* in the turbot primary hepatocytes. The results are expressed as the mean ± standard deviation (n = 4). ‘*’ means significantly different between two group (*P* < 0.05). The different letters are significantly different (*P* < 0.05).

The mRNA levels of *tnf-α*, *il-1β*, *il-6*, *nf-κb*, *myd88*, *cd83* and *tlr-2* in each group significantly increased after LPS incubation compared with pcDNA3.1 group (*P* < 0.05). The mRNA levels of *tnf-α*, *il-6*, *nf-κb*, *cd83* and *tlr-2* significantly decreased in pcDNA3.1-*foxo3*+LPS group compared with pcDNA3.1+LPS group (*P* < 0.05). The mRNA levels of *il-1β* and *myd88* decreased in pcDNA3.1-*foxo3*+LPS group compared with pcDNA3.1+LPS group, but there was no significant difference between the two groups (*P* > 0.05). The mRNA levels of *jnk* remained unchanged among the three groups (*P* > 0.05) (**Figure 9**).

**Figure 10:**
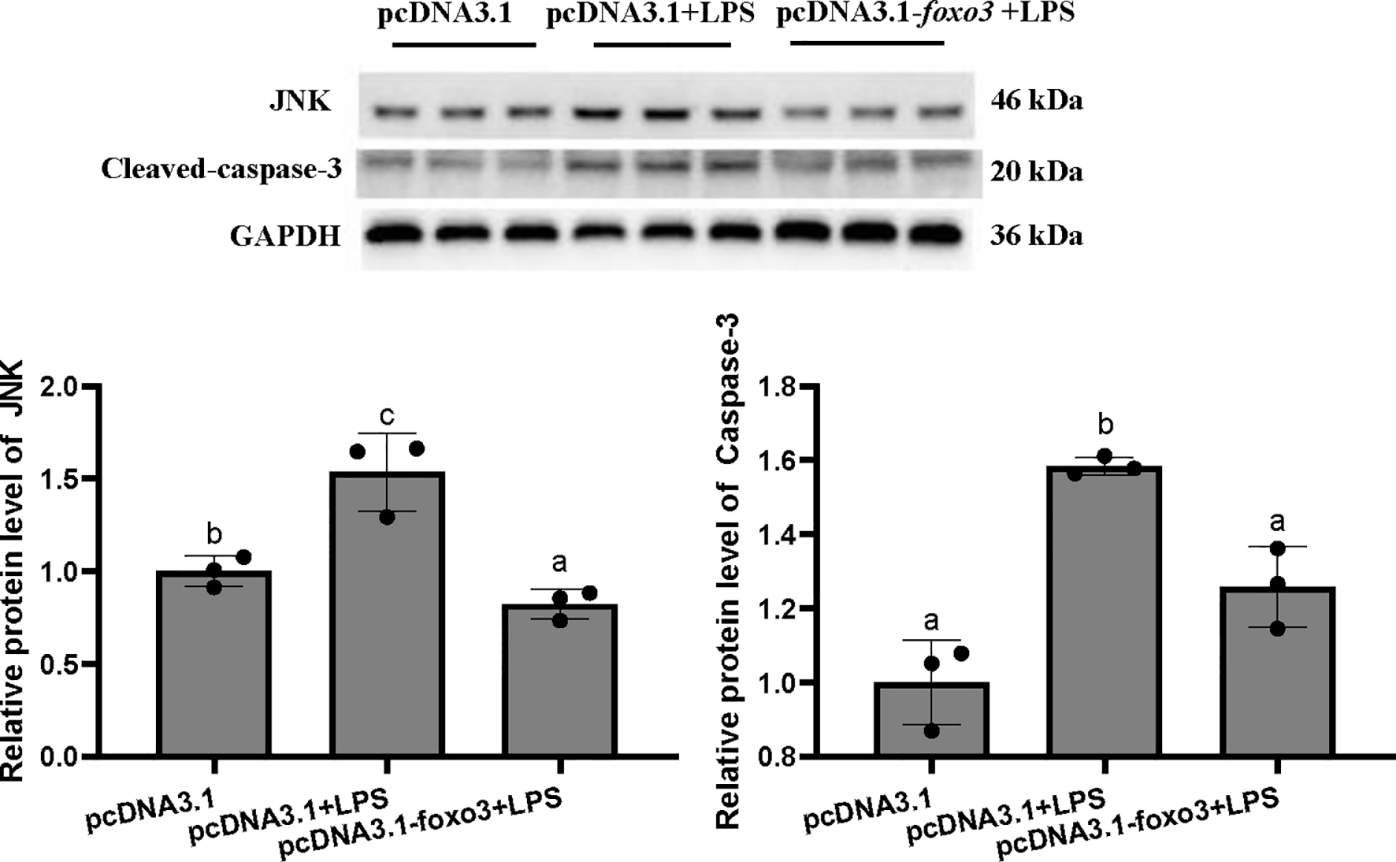
Protein levels in turbot hepatocytes. The relative protein abundances of JNK and cleaved- caspase-3 in hepatocytes were measured by western blot and expressed as relative expression values to those in pcDNA3.1 group. Values are represented as means the mean ± standard deviation (n = 3). Values with different letters mean significantly difference (*P* < 0.05).

The protein levels of JNK and cleaved-caspase-3 significantly increased after LPS incubation compared with pcDNA3.1 group (*P* < 0.05). The protein levels of JNK and cleaved-caspase-3 significantly decreased in pcDNA3.1-*foxo3*+LPS group compared with pcDNA3.1+LPS group (*P* < 0.05) (**Figure 10**).

### Inflammation-Related Genes, Enzyme Activity and Proteins With Foxo3 Overexpressed *In Vivo*

**Figure 11:**
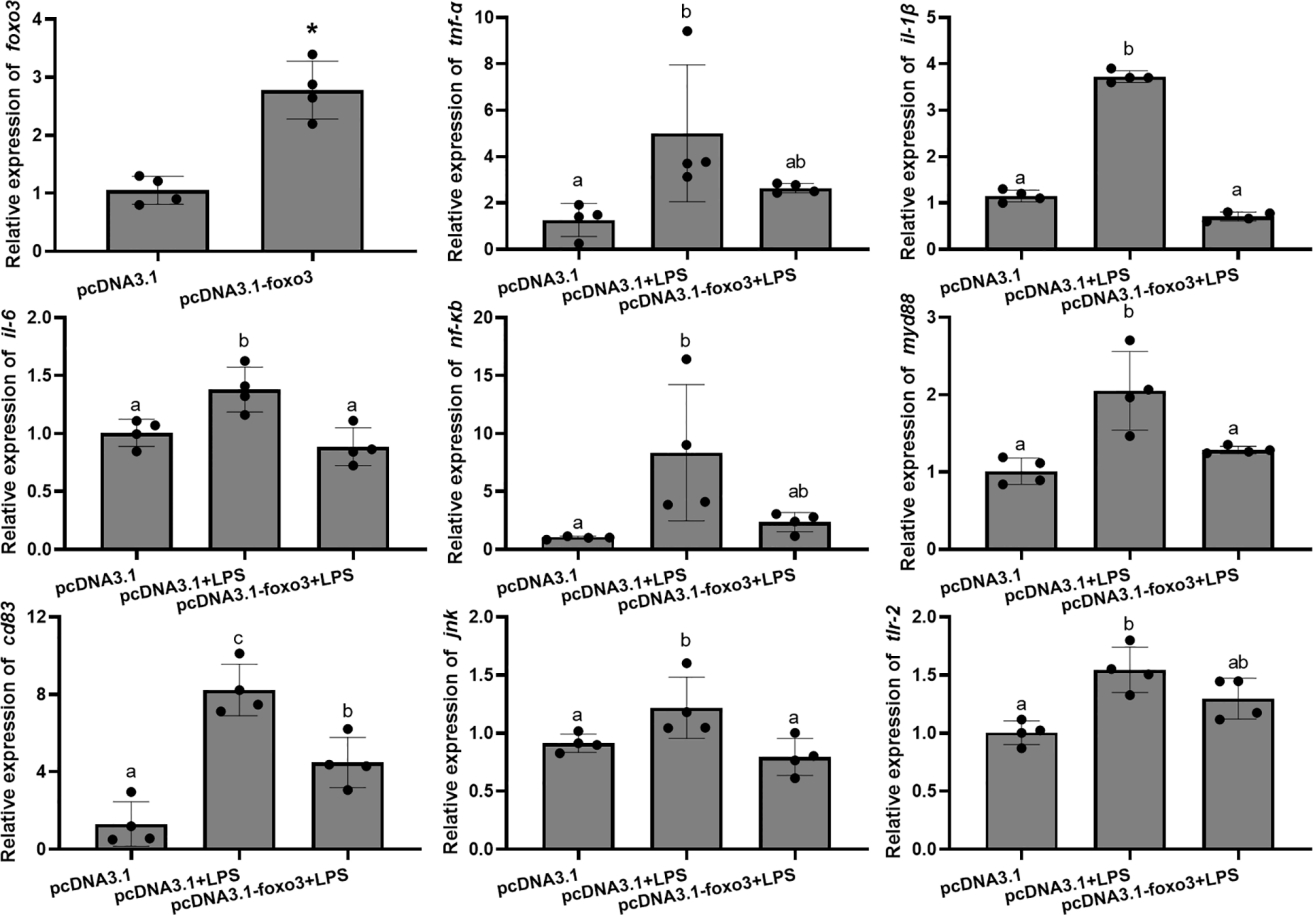
Relative mRNA levels of *foxo3*, *tnf-α*, *il-1β*, *il-6*, *nf-κb*, *myd88*, *cd83*, *jnk*, *tlr-2* in turbot liver. The results are expressed as the mean ± standard deviation (n = 4). ‘*’ means significantly different between two group (*P* < 0.05). The different letters are significantly different (*P* < 0.05).

To investigate the involvement of FoxO3 factor in LPS-induced liver inflammation *in vivo*, turbots injected with overexpression plasmid *in vivo*. The result indicated that pcDNA3.1-*foxo3* upregulated *foxo3* expression in turbot liver cells (increasing about 300%) (**Figure 11**). The mRNA levels of *tnf-α*, *il-1β*, *il-6*, *nf-κb*, *myd88*, *cd83*, *jnk*, and *tlr-2* significantly increased after LPS injection compared with pcDNA3.1 group (*P* < 0.05). The mRNA levels of *il-1β*, *il-6*, *myd88*, *cd83* and *jnk* significantly decreased in pcDNA3.1-*foxo3*+LPS group compared with pcDNA3.1+LPS group (*P* < 0.05). The mRNA levels of *tnf-α*, *nf-κb* and *tlr-2* decreased in pcDNA3.1-*foxo3*+LPS group compared with pcDNA3.1+LPS group, but there was no significant difference between the two groups (*P* > 0.05) (**Figure 11**).

**Figure 12:**
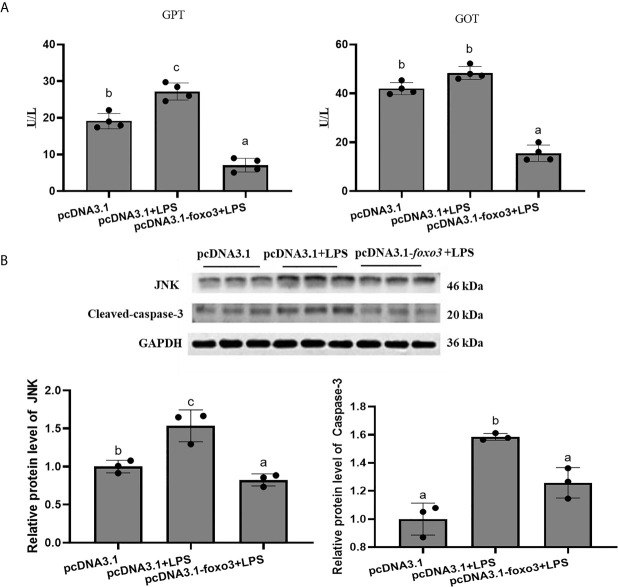
**(A)** The levels of glutamate pyruvate transaminase (GPT) and glutamate oxaloacetate transaminase (GOT) in serum. Values are represented as the mean ± standard deviation (n = 4). Values with different letters mean significantly difference (*P* < 0.05). **(B)** Protein levels in turbot liver. The relative protein abundances of JNK and cleaced-caspase-3 in turbot liver were measured by western blot and expressed as relative expression values to those in pcDNA3.1 group. Values are represented as the mean ± standard deviation (n = 3). Values with different letters mean significantly difference (*P* < 0.05).

The results of GPT and GOT in serum showed that the level of GPT significantly increased after LPS injection compared with pcDNA3.1 group (*P* < 0.05) (**Figure 12A**). The level of GOT increased after LPS injection compared with pcDNA3.1 group, but there was no significant difference between the two groups (*P* > 0.05) (**Figure 12A**). The levels of GPT and GOT in serum significantly decreased in pcDNA3.1-*foxo3*+LPS group compared with pcDNA3.1+LPS group (*P* < 0.05) (**Figure 12A**).

The protein levels of JNK and cleaved-caspase-3 significantly increased after LPS injection compared with pcDNA3.1 group (*P* < 0.05). The protein levels of JNK and cleaved-caspase-3 significantly decreased in pcDNA3.1-*foxo3*+LPS group compared with pcDNA3.1+LPS group (*P* < 0.05) (**Figure 12B**).

## Discussion

The *foxo3* belongs to the family of forkhead box O transcription factors and has emerged as an important role in immune regulation in mammals’ liver (17, 29–31). In the present study, the *foxo3* was cloned and characterized in turbot for the first time. The *foxo3* mRNA was identified and the full-length ORF of *foxo3* was 1998 bp, encoding peptides of 665 amino acids. In mammals, *foxo3* is widely expressed in various tissues, including skeletal muscle, heart muscle, kidney, liver, pancreas, thymus and nervous system (32). In the present study, *foxo3* was expressed in all the 12 analyzed tissues and the relative expression was highest in muscle, following by adipose tissue and skin. The relative expression of *foxo3* was not the highest in turbot liver. However, the liver can produce defense regulators and mediators and has recently led to its perception as an important organ of innate immunity in mammals (33). In addition, the role of *foxo3* in liver immunity and injury has been studied in many researches in other species (17, 30, 34, 35). Moreover, in the study of catfish, it was found that after *E. ictaluri* infection, *foxo3* was significantly upregulated in intestine, suggesting its involvement in responses bacterial infection in catfish intestine (20). In the present study, the relative expression of *foxo3* in liver was higher in intestine. Therefore, it was suspected that FoxO3 in liver may also play a crucial role in immune responses. This is the first time to report on the localization of *foxo3* in fish. From the results of immunofluorescence, *foxo3*, as a transcription factor, expressed in cytoplasm as well as in nucleus. Similar results were found in the study of human placenta and fetal membranes (36). In mammals, it was confirmed that the functions of *foxo3* were related to changes in its cellular localization (37). However, further researches are needed in fish

LPS-induced liver injury has been used as experimental models to analyze the mechanism of endotoxin-induced acute liver injury (38, 39). LPS induces the release of numerous proinflammatory cytokines, including interleukin-1 (*il-1*), *il-6*, and tumor necrosis factor (*tnf*) both in mammals and fish (6, 40–42). Hence, LPS incubated *in vitro* and injected *in vivo* were used to construct inflammatory models. In the present study, the relative expressions of *il-1β*, *il-6* and *tnfα* increased after LPS administration both *in vivo* and *in vitro*. Meanwhile, knockdown of *foxo3* led to significant elevating of these mRNA levels, while the *foxo3* overexpression significantly decreased these levels. This is consistent with the previous study, in which *foxo3* overexpression in intestinal epithelial cells caused a significant decrease in the gene expressions of *il-1* and *il-6* (13). In mice, silence of *foxo3* increased the gene expressions of *il-1β*, *il-6* and *tnfα*, but overexpressing of *foxo3* attenuated liver failure by decreasing the mRNA levels of those genes (16). Therefore, the conclusion can be drawn that *foxo3* can suppress inflammation by regulating proinflammatory cytokines.

GPT and GOT in serum are markers of hepatic damage, and the GPT and GOT levels in serum were increased in liver failure patients (43). In addition, when fish were exposed to environmental toxins, the serum levels of GPT and GOT increased significantly (44). Moreover, when juvenile red sea bream (*Pagrus major*) fed with the diet with excessive amounts of nucleoside by-products, the serum level of GPT increased significantly, which consistent with liver damage (45). Therefore, increased GPT and GOT levels were considered to indicate hepatocyte injury or liver damage in fish. In the present study, when turbots were injected with LPS (2.5 μg/g), the levels of GPT and GOT were increased. The results showed that LPS could cause liver damage in turbot. When the turbots were injected with si*foxo3* for 24 h before the turbots were injected with LPS, the levels of GPT and GOT in serum increased compared with the NC+LPS group. It was indicated that inhibition of *foxo3 in vivo* increased LPS-activated liver damage in turbot. However, when the turbots were injected with pcDNA3.1-*foxo3* for 24 h before the turbots were injected with LPS, the levels of GPT and GOT in serum decreased compared with the pcDNA3.1+LPS group. It was indicated that overexpression of *foxo3 in vivo* relieved LPS-activated liver injury in turbot. Therefore, it is suggested that *foxo3* can relieve liver damage induced by LPS.

JNK has been implicated in involving in liver damage (46) and triggering the activation of *foxo3* in response to immune stress (47, 48). In breast cancer cells, cardamonin induces apoptosis *via* activation of the JNK/FoxO3 pathway (49). According to the report of Yang et al. (50), FoxO3 *via* ROS/JNK/FoxO3 pathway plays a critical role to function as negative regulator associating with deoxynivalenol-induced cytotoxicity, with the potential extending to other substances (50). Therefore, there is a close relationship between JNK and FoxO3 when they play the role in immune regulation. In the present study, knock down of *foxo3* could not change the gene expressions of *jnk*, but increased the protein levels of JNK both *in vivo* and *vitro*. The results indicated that JNK was affected by *foxo3* at the protein level rather than the gene expression level in turbot liver cells. Furthermore, it has been confirmed that FoxO3/JNK pathway can regulate apoptosis (51–53), so the protein levels of cleaved-caspase-3 were detected in the present study. The present results showed that the protein levels of cleaved-caspase-3 had not been changed after *foxo3* knocking down. Interestingly, the protein levels of JNK and cleaved-caspase-3 showed the same trend when overexpression of *foxo3* both *in vitro* and *in vivo*, which revealed that *foxo3* was a negatively regulated transcription factor in the JNK and caspase-3 mediated pathways. Those results indicated *foxo3* regulated immune function through both caspase-3 dependent and independent in JNK/caspase-3 pathway.

It was reported that *tlr-2* was a major mediator of the inflammatory response, which associated with inflammation and liver injury tightly (54–56). Ma et al. (57) reported that *tlr-2* could activate the *nf-κb* pathway to regulate inflammatory response in a *myd88*-dependent pathway (57). Interestingly, the phenomenon was confirmed in the present study, in which the mRNA levels of *tlr2*, *myd88* and *nf-κb* were up-regulated after LPS incubating. In addition, knockdown of *foxo1* and *foxo3* increased endogenous *tlr-2* mRNA levels. It was suggested that *foxo1/3* suppresses *tlr-2* in keratinocytes (58). In the present study, the results showed that knockdown of *foxo3* both *in vitro* and *in vivo* could up-regulate the mRNA levels of *tlr-2*, *myd88* and *nf-κb*. Moreover, overexpression of *foxo3* both *in vitro* and *in vivo* could reduce the mRNA levels of *tlr2*, *myd88* and *nf-κb*. These results indicated that LPS could activated *tlr-2*/myd88/*nf-κb* pathway and *foxo3* inhibit LPS-induced inflammation through this signal pathway.

To sum up, the full-length ORF of *foxo3* sequence was cloned from turbot. FoxO3 overexpression can inhibit LPS-induced inflammation *in vivo* and *vitro* by decreasing proinflammatory cytokines, the levels of GOT and GPT as well activating JNK/caspase-3 and *tlr-2/myd88/nf-κb* pathways. FoxO3 knocking down can activate LPS-induced inflammation through above signaling pathways *in vivo* and *vitro*. The research of FoxO3 in liver immunity in turbot enriches the studies on immune regulation in fish, and provides theoretical basis and molecular targets for solving liver inflammation and liver injury in fish.

## Data Availability Statement

The datasets presented in this study can be found in online repositories. The names of the repository/repositories and accession number(s) can be found in the article/**Supplementary Material**.

## Ethics Statement

The animal study was reviewed and approved by Institutional Animal Care and Use Committee of Ocean University of China. Written informed consent was obtained from the owners for the participation of their animals in this study.

## Author Contributions

MP: Data curation, analysis and writing original draft. JL, DH, YG, KL, and MY: Data curation. WG and QX: Conceptualization. WZ: Conceptualization, funding acquisition, methodology, supervision, writing review and editing. KM: Methodology. All authors contributed to the article and approved the submitted version.

## Funding

This study was financially supported by the National Key R & D Program of China (grant number: 2019YFD09002 00, 2018YFD0900400).

## Conflict of Interest

The authors declare that the research was conducted in the absence of any commercial or financial relationships that could be construed as a potential conflict of interest.
