# Supplementary material for: FoxO3 Modulates LPS-Activated Hepatic Inflammation in Turbot (Scophthalmus maximus L.)
Source: Front Immunol. 2021 Jul 1;12:679704. doi: 10.3389/fimmu.2021.679704 (PMC8281027; doi:10.3389/fimmu.2021.679704)

**Figure legends**

**Supplementary Figure 1.** The comparison of amino acid (aa) sequences of FoxO3 between human (19aa~67aa) and turbot (18aa~59aa) in Clustal Omega website (<https://www.ebi.ac.uk/Tools/msa/clustalo/>), the similarity of two sequence is 71.7%.

**Supplementary Figure 2.** The whole gel to check the molecular weight of FoxO3 (96 kDa) in turbot liver. The first lane was the marker. The other three lanes were the same sample of turbot liver protein with three replicates (30 μg protein per lane), respectively.

**Supplementary Figure 3.** The whole gels of GFP (27 kDa) to determine whether the pcDNA3.1-EGFP can transfer into tissues by intraperitoneal injection in turbot. A, B and C were the different tissues (A: intestine; B: kidney; C: liver) of turbot injected with pcDNA3.1- EGFP intraperitoneally. D was the liver of turbots injected with PBS intraperitoneally. The primary monoclonal was mouse anti-jellyfish antibody for GFP (1:1000, AF609, Beyotime, China). The secondary antibody was HRP-labeled goat anti-mouse lgG (1:5000, A0216, Beyotime, China) at 1:5000.

**Supplementary Figure 4.** The whole gels to check the molecular weight of JNK (46 kDa), GAPDH (36 kDa) and Caspase3 (20 kDa) in turbot liver. The first lane was the marker. The other three lanes were the same sample of turbot liver protein with three replicates (30 μg protein per lane), respectively.

**Supplementary Figure 1.**


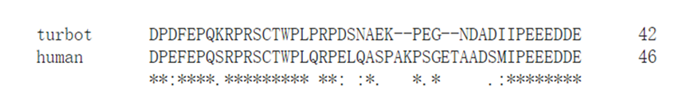


**Supplementary Figure 2.**


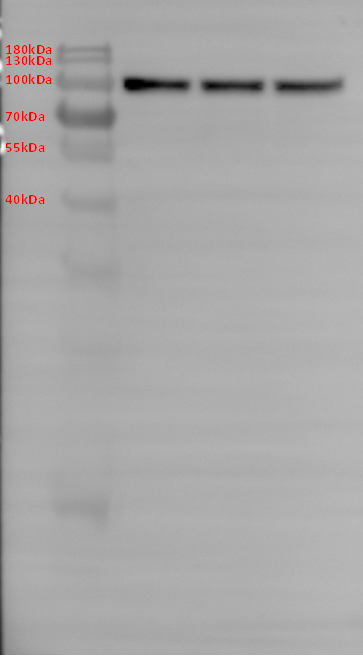


**Supplementary Figure 3.**


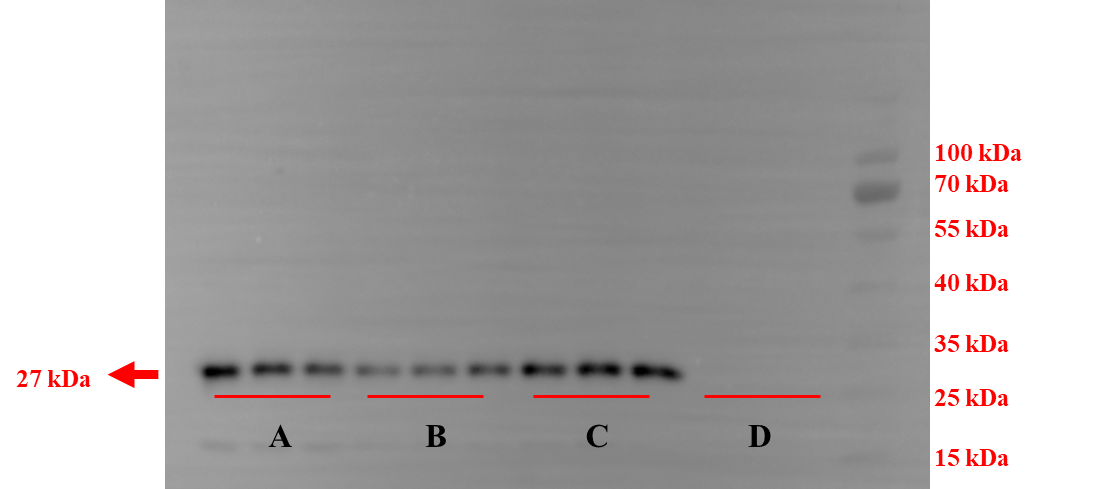


**Supplementary Figure 4.**


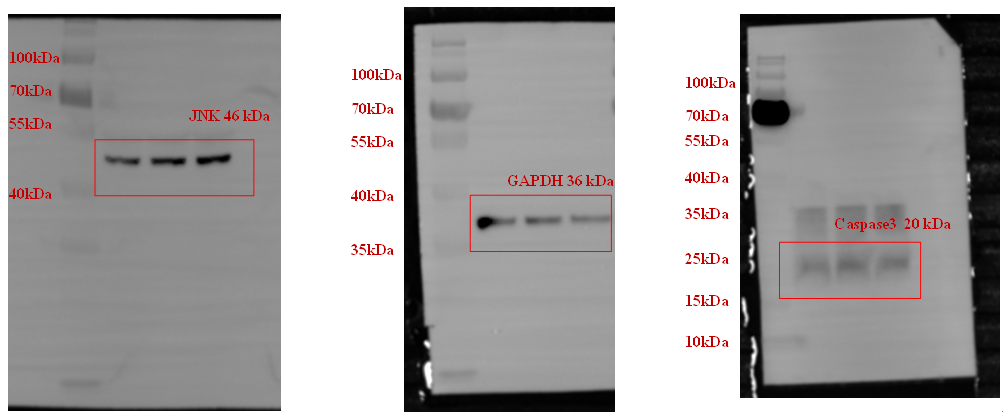

Supplement: Supplementary file 1 [file DataSheet_1.doc]
